# Evidence for genes associated with the ability of *Mycobacterium avium* subsp. *hominissuis* to escape apoptotic macrophages

**DOI:** 10.3389/fcimb.2015.00063

**Published:** 2015-08-25

**Authors:** Luiz E. Bermudez, Lia Danelishvili, Lmar Babrack, Tuan Pham

**Affiliations:** ^1^Department of Biomedical Sciences, College of Veterinary Medicine, Oregon State UniversityCorvallis, OR, USA; ^2^Department of Microbiology, College of Science, Oregon State UniversityCorvallis, OR, USA; ^3^Program of Molecular and Cell Biology, Oregon State UniversityCorvallis, OR, USA; ^4^Biochemistry Program, College of Science, Oregon State UniversityCorvallis, OR, USA

**Keywords:** *M. avium*, macrophages, apoptosis, exit from macrophages, MAV_2235, MAV_2122, MAV_4564, TaTC

## Abstract

*Mycobacterium avium* subsp. *hominissuis* (MAH) is an environmental bacteria that infects immunocompromised humans. MAH cases are increasing in incidence, making it crucial to gain knowledge of the pathogenic mechanisms associated with the bacterium. MAH infects macrophages and after several days the infection triggers the phagocyte apoptosis. Many of the intracellular MAH escape the cell undergoing apoptosis leading to infection of neighboring macrophages. We screened a transposon bank of MAH mutants in U937 mononuclear phagocytes for the inability to escape macrophages undergoing apoptosis. Mutations in genes; MAV_2235, MAV_2120, MAV_2410, and MAV_4563 resulted in the inability of the bacteria to exit macrophages upon apoptosis. Complementation of the mutations corrected the phenotype either completely or partially. Testing for the ability of the mutants to survive in macrophages compared to the wild-type bacterium revealed that the mutant clones were not attenuated up to 4 days of infection. Testing *in vivo*, however, demonstrated that all the MAH clones were attenuated compared with the wild-type MAC 104 in tissues of mice. Although the mechanism associated with the bacterial inability to leave apoptotic macrophages is unknown, the identification of macrophage cytoplasm targets for the MAH proteins suggest that they interfere either with protein degradation machinery or post-translation mechanisms. The identification of tatC as a MAH protein involved in the ability of MAH to leave macrophages, suggests that secreted effector(s) are involved in the process. The study reveals a pathway of escape from macrophages, not shared with *Mycobacterium tuberculosis*.

## Introduction

*Mycobacterium avium* subsp. *hominissuis* (MAH) is an environmental bacterium that can infect the human host causing both lung pathology and disseminated disease (Falkinham, 1996). In patients, MAH usually is associated with opportunistic infections, although in many reports it appears also to cause disease in immunocompetent individuals (McGarvey and Bermudez, 2002; Turenne et al., 2007). In some cases recently described, specific immunodeficiencies besides AIDS were identified as underlying conditions to explain MAH disseminated behavior (Dominici et al., 2012).

MAH infects macrophages and is able to replicate intracellularly (Hussain et al., 1999). Macrophages respond to MAH infection by upregulating bactericidal mechanisms, however without much success as exemplified by the inability to kill MAH by producing superoxide anion, nitric oxide, or by the induction of autophagy (Bermudez and Young, 1989; Bermudez, 1993). In addition MAH is resistant to bactericidal proteins encountered in phagocytes such as cathelicidin (Motamedi et al., 2014). The bacterium inhibits the acidification of the mycobacterial vacuole, as well as the fusion of phagasomes with lysosomes (Sturgill-Koszycki et al., 1994) and delivery of toxic elements to the vacuole environment. It appears that as the last resource, to be able to eliminate the pathogen, *M*. *avium*-infected macrophages undergo apoptosis (Early et al., 2011). Several studies in the *Mycobacterium tuberculosis* field have demonstrated that bacterium in many occasions can inhibit macrophage-triggered apoptosis (Danelishvili et al., 2003; Velmurugan et al., 2007), and recent findings have also shown, at least in one model, that *M*. *avium* subsp. *paratuberculosis* infection is associated with suppression of apoptosis (Kabara and Coussens, 2012). Apoptosis, although partially effective, also does not seem to be the predominant mechanism of killing in MAH infected macrophages. In fact, the existing evidence appears to indicate that MAH has evolved adaptive approaches to cope with apoptosis of macrophages, being capable of exiting apoptotic cells and infect, surrounding macrophages (Kabara and Coussens, 2012). It is interesting that has been observed a correlation between strain-dependent effect with *M. tuberculosis*, and virulent strains of *M. tuberculosis* in mice. The increasingly, virulent strains induced more necrosis and less apoptosis of macrophages (Park et al., 2006). In addition, work by Lee et al. (2006) demonstrated that infection of macrophages with *M.tuberculosis* using a high MOI resulted in cell death by pyroptosis, while infection low MOI inhibited apoptosis.

Although not all bacteria inside apoptotic macrophages are successful in escaping the environment, some appear to “find” the cytoplasmic membrane and exit the cell (Kabara and Coussens, 2012). Others stay inside apoptotic cells (spherocytosis) and are subsequently ingested by other macrophages, with unknown outcome.

Many diverse pathogens have been shown to trigger apoptosis of the host cell as a mechanism of escaping the host response (Monack et al., 1997, 2001). For instance, *Yersinia enterocolitica* induce macrophage apoptosis to avoid uptake (Monack et al., 1997), and *Salmonella enterica Typhimurium* has two distinct mechanisms to kill host cells by apoptosis (Monack et al., 2001). Recently, we have described a new form of host cell apoptosis induced by MAH, which is only observed upon entry into the “secondary-infected host macrophages” (Early et al., 2011). Thus far, the mechanism(s) associated with it is unknown.

Once the host cell initiates the process of apoptosis, the intracellular bacteria need to sense it, in order to forge the escape from the dying cell. In fact, by carefully observing macrophages infected with MAH, we described that many of the intracellular MAH, although not all the intracellular bacteria, exit apoptotic macrophages (Kabara and Coussens, 2012). These bacteria are then capable of infecting other cells and causing the spread of the infection.

In order to determine the genetic components important for the MAH strategy of leaving apoptotic macrophages, we developed a screening system using U937 phagocytes and evaluated approximately 3000 transposon clones using it. Among the mutants screened, four were incapable of escaping apoptotic macrophages. In the current study we begin to describe the mechanisms associated with MAH's ability to exit apoptotic macrophages.

## Materials and methods

### Bacteria and growth conditions

MAH strain 104 a clinical isolated first obtained from the blood of an AIDS patient, was used for the described assays. MAH 104 was cultured on Middlebrook 7H11 agar supplemented with oleic acid, albumin, dextrose, and catalase, OADC (Hardy Diagnostics, Santa Maria, CA) for 10–12 days. After, pure colonies were obtained, and the bacteria were grown to logarithmic phase in Middlebrook 7H9 broth. *M*. *avium* transposon library (strain 104) was created as previously reported (Motamedi et al., 2014). Clones were stored individually. For the library screening experiments, clones were grown in presence of 400 μg/ml of kanamycin, harvested, and used in the assay.

### Macrophages

U937 monocytic cell line was used to screen the transposon bank of mutants. U937 were grown in RPMI-1640 supplemented with 5% fetal bovine serum (Sigma, St.Louis, MO). Approximately 10^5^ cells were added per well of a 24-well tissue culture plate and incubated in the presence of 0.5 μg/ml of phorbol myristate acetate (PMA), for 12 h to mature the mononuclear phagocytes. Once the initial screening provided mutants, the selective mutant clones were then tested again using THP-1 mononuclear cells. THP-1 cells were seeded as described for U937 and exposed to 0.5 μg/ml PMA (Sigma Chemical Co., St. Louis, MO) for 4 h to mature. Human monocyte-derived macrophage monolayers obtained from blood bank (IRB approval # 2120) were prepared as previously described (Early and Bermudez, 2011). Each monolayer contained approximately 2 × 10^5^ cells. Infection of macrophages monolayers were carried out as previously reported (Early and Bermudez, 2011) at MOI of 10. Bacteria were allowed to infect cells for 1 h and then the bacteria in the supernatant were removed by washing. The number of phagocytosed bacteria was determined by lysing the monolayer with sterile water, and plating the homogenate onto 7H10 agar plates with OADC (oleic acid, albumin, dextrose, catalase). The percent of macrophage viability was determined using trypan blue as previously described (Early and Bermudez, 2011). Only monolayers with greater than 90% viability were used for the experiments.

### Screening of the transposon library

An *M. avium* 104 transposon library was created as described previously (Motamedi et al., 2014). Approximately 3000 clones (60% of the genome) were evaluated individually for the inability to leave macrophages in culture. It has been shown that one can use U937 macrophages to accurately determine apoptosis/anti-apoptotic effect of *M*. *tuberculosis* and MAH. Nine-six-well tissue culture plates were seeded with U937 macrophages (1 × 10^5^ cells/well) and matured by the addition of phorbol myristate acetate (PMA, Sigma, St. Louis, MI) for 24 h. Mature macrophage monolayers were then washed once with RPMI-1640 medium and incubated for 24 h with RPMI-1640 supplemented with 10% fetal bovine serum. At this point, each well was infected with 10^5^ bacteria belonging to different clone of MAH. The plates were followed visually for 7 days. As described previously, the wild-type MAH 104 monolayers start detaching from day 4 of culture, and approximately 100% of the detaching macrophages are apoptotic (Early and Bermudez, 2011). Extracellular bacteria began to be observed in the monolayers as described. By following the U937 monolayers for 7 days, it became clear that in selected monolayers, fewer bacteria were seen in the extracellular environment. Those monolayers were chosen (23 out of 3000 clones) and submitted to a second screening in U937 cells. Following confirmation, the clones were evaluated using human monocyte-derived macrophages, and from the 23 clones, four were selected due to the marked phenotype. The final four clones showed similar phenotypes in THP-1 macrophages and human monocyte-derived macrophages. Southern blot confirmed that all the mutants only had one transposon.

### Invasion and survival assays

Macrophage invasion and survival assays were carried out as described previously (Li et al., 2010). THP-1 macrophages (1 × 10^5^/monolayer) were infected with 10^6^ bacteria for 1 h. Then, the monolayers were lysed with sterile water and 0.25%/SDS as described (Li et al., 2010). The obtained lysate was serially diluted and plated onto 7H11 agar plates. The remaining monolayers were followed for 4 days and lysed. The viable intracellular bacteria were diluted and plated onto 7H11 agar, to determine if the bacteria grew or not inside macrophages. The CFU/ml of lysate was then compared with the CFU/ml of the lysate at time 0 (1 h after infection) and with the number of bacteria (MAH 104 wild-type control) at 4 days after infection.

### Gene sequencing

To determine the genes inactivated in the four MAH clones, bacterial DNA was purified and sequences were obtained by using the method as previously described (Danelishvili et al., 2014). Primers were designed for the four genes and amplification was attempted using the following protocol: The unknown sequence was amplified using PCR primers for the transposon as previously described (Li et al., 2010). The product was purified and directly sequenced using an ABI 373 DNA sequencer with a FAS directory terminator cycle sequencing kit (Perkin-Elmer, CA). Only sequences that contained the transposon were considered. The obtained sequences were mapped to the *M*. *avium* 104 genome sequence (NCBI database) and *M*. *tuberculosis* H37Rv (TubercuList) genome sequence adding to the blast program.

### Protein-protein interaction

The protein-protein interaction assays were performed as previously described (Danelishvili et al., 2014). Because it is possible that the MAH proteins associated with the ability of the bacterium to exit the infected macrophage interacted with macrophage proteins, we purified the proteins and submitted them to a protein-protein interaction. Because results with the TatC transport systems would be challenging to interpret, we decided not to include the protein in the assays.

The bacterial proteins were cloned in the two-hybrid system and the ability to interact was determined using a human macrophage library as described (Danelishvili et al., 2014). *M. avium* genes were cloned in frame with the GAL4 DNA binding domain of pGBKT7 and resultant vectors were transformed into *Saccharomyces cerevisiae* strain Y2HGold following the manufacturer's recommendations (Clontech). The Matchmaker Gold Yeast Two Hybrid Universal Human Library was purchased from Clontech. The library was created in fusion with GAL4 activation domain of pGADT7 vector and stored in Y187 yeast strain. The interaction between pGBKT7-53 and pGADT7-T served as a positive control, whereas pGBKT7-lam and pGADT7-T was used as a control for negative interaction. One milliliter of the library was combined with 4 ml of a bait yeast strain and grown in 2xYPDA liquid medium containing 50 μg/ml kanamycin at 30°C for 24 h with slow shaking (50 rpm). Zygotes were plated on Double (SD–Leu/–Trp), Triple (SD–His/–Leu/–Trp), and Quadruple Dropout (SD–Ade/–His/–Leu/–Trp) agar plates with or without 20 mg/ml of X-α-Galactosidase and 125 ng/ml Aureobasidin. Colonies that turned blue were PCR amplified using the Matchmaker Insert Check PCR Mix 2 (Clontech) and resulting products were sequenced at the CGRB facility of Oregon State University. The positive interaction was established with the reverse screening where identified human cDNAs were cloned into pGBKT7 vector and bait *M. avium* genes (MAV_2120, MAV_2235, and MAV_4563) were cloned into pGADT7.

### Co-immunoprecipitation

The CSN5 protein was expressed in the pET6xHN-C vector (Clontech) in *E. coli* and purified with a His-column according to the manufacturer's protocol (Clontech). Alternatively, *M. smegmatis* expressing Flag:MAV_2120 and Flag:MAV_4563 in pMV261 were lysed by mechanical disruption. The purified CSN5 protein was incubated with *M. smegmatis* cell lysate expressing MAV_2120 and MAV_4563 proteins. After overnight incubation, at 4°C, samples were loaded into His-columns, washed and eluted. Proteins of interest were visualized with western blotting using 6xHN and Flag antibodies.

### Bioinformatics analysis

The DNA sequences obtained in the mutants were identified by matching the sequences with the MAC104 sequence in the database (Danelishvili et al., 2014). The MAH gene sequence was then analyzed using NCBI database for homologous genes and for conserved domains. Programs were used to look for specific motifs in the proteins. Bioinformatic analysis of potential operons was performed using Softberry software (Softberry, Mt Kisko, NY).

### Gene expression

To determine whether the *M*. *avium* 104 genes were expressed in the course of bacterial infection of macrophages, the primers described above were used to amplify cDNA obtained from intracellular *M*. *avium* at time points 1, 3, 5, and 7 days following infection. Bacterial RNA was purified using protocol previously described (Danelishvili et al., 2014). Briefly, intracellular bacteria were obtained by differential centrifugation of lysed THP-1 macrophages (10^8^ cells infected with 1 × 10^8^ bacteria) with 2 ml of Trizol (Invitrogren, Carlsbad, CA). The mixture Atlas Pure Total RNA labeling system (Clontech Laboratories, Palo Alto, CA) was used according to the manufacturer's instructions. The RNA was then treated with DNAase for 30 min at 37°C followed by phenol-chloroform extraction and precipitation with ethanol. The RNA was then run on 1% denaturing agarose gel and quantified by UV spectrophometer at 260/280 min. First-strand cDNA was synthesized using random hexamer primers and the superscript cDNA synthesis kit (Invitrogen, Carlsbad, CA) according to the manufacturer's instructions.

### Video microscopy

To confirm the phenotypes of the mutants, THP-1 monolayers (1 × 10^5^ cells) were established in cover slips and transferred to home-made sterile chambers for video microscopy. Macrophage monolayers were infected with MOI 10 or 1 with *M*. *avium* 104 or mutants. At several times after infection, monolayers were subjected to time lapse video microscopy on a Nikon microscope and the video images were collected with an Optonics DEI_750 camera. The experiments were repeated at least 3 times and 50 macrophage/events were observed before a specific data collection took place.

### Complementation of phenotype

Complementation of the different mutants (MAV_ 2235, MAV_ 2410, MAV_2463, and MAV_2120) were carried out using published methodology (Li et al., 2005). Briefly, the genes were amplified from the genome DNA and the PCR product was cloned onto pFJS8 vector containing the L5 promoter upstream of the cloning site. The plasmid was then used to transform the mutants. Clones containing the plasmid were selected in presence of the kanamycin and apramycin using qRT-PCR and Western-blot. All four genes were expressed.

### Mice infection

The study was approved by the IACUC under the protocol number 4396. C57BL/six black mice (10 mice/group plus six mice in baseline control group) were infected either with the wild-type bacteria, the mutant or the complemented mutant I.V. as previously described (Li et al., 2005). At the second day, six mice were harvested in the control group to determine the initial tissue load (spleen). The other 10 mice in the experimental group were harvested at the end of week 3 of infection. The spleen was obtained, homogenized as previously described (Li et al., 2005) and the homogenate was then serially diluted and plated onto 7H11 agar with carbenicillin, amphotericin B, trimethoprim-sulfametoxazol, and polymyxin B (Li et al., 2005). The plates were incubated for 20 days and then counted to determine the bacterial load. The difference in bacterial CFU per gram of tissue between day 2 and day 21 was compared for all groups.

### Statistical analysis

*In vitro* experiments were repeated at least three times and the comparison between experimental groups were analyzed using the Prism software (GraphPad, San Diego, CA) using the Student's *t*-test. In addition One-Way ANOVA was used to calculate the results of the mice study.

## Results

### Screening for clones that were unable to exit apoptotic macrophages

Past work has demonstrated that once macrophages are infected with MAH for 4 or more days, they begin undergoing apoptosis, followed by many bacteria leaving the cell (Early et al., 2011). This observation has been confirmed with several strains of MAH. Briefly, MAH-infected macrophages at an MOI of 10, develop apoptosis after 4 days of infection of U937 phagocytes, and can be identified by detaching from the monolayer (Danelishvili et al., 2003). While monolayers infected with the wild-type MAH after 4 days may contain extracellular bacteria, mutants incapable of escaping the infected phagocytes could not be observed in the tissue culture media, nor be cultured out of the culture supernatant.

**Figure 1:**
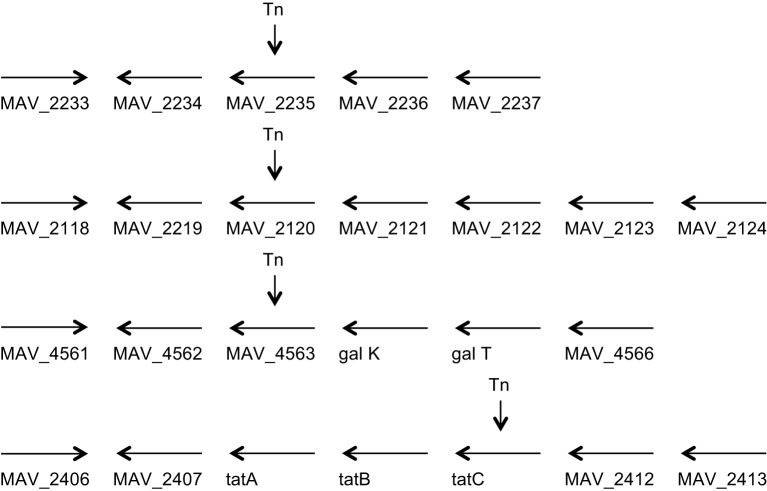
**Genome organization of the four genes identified to be associated with the ability to escape macrophages**. MAV_2235 encodes for an adenylate cyclase, MAV_2120 encodes for a divalent cation transport, MAV_4563 encodes for a protein of unknown function, and MAV_2410 encodes for the transport protein tatC.

We used this specific characteristic to screen for MAH clones that did not exit macrophages (U937 cells) 4 days after infection. By examining 3000 clones we were able to visually select 23 clones, that were then re-examined in THP-1 macrophages and monocyte-derived macrophages. Four clones, not capable of leaving macrophages upon apoptosis were ultimately selected for further investigation (Figure 1). Clone VLF4/14 had transposon inserted on MAV_2235, clone VLF3/18 had the gene MAV_2120 interrupted, the clone VLF4/28 had the gene MAV_4563 interrupted and the clone VLF4/34 had a transposon in MAV_2410 (Table 1).

**Table 1:** **Ability of MAH to escape U937 macrophages**.

**Strain/gene**	**No. of bacteria outside macrophages**		
	**24 h**	**72 h**	**120 h**
MAC 104 WT	Undetectable	135 ± 26	3.2 ±0.3 × 10^3^
MAV_2235	Undetectable	Undetectable	Undetectable
MAV_2235c	Undetectable	96 ± 41	1.9 ±0.6 × 10^3^
MAV_2120	Undetectable	Undetectable	Undetectable
MAV_2120c	Undetectable	Undetectable	8.9 ±0.5 × 10^2^
MAV_2410	Undetectable	Undetectable	Undetectable
MAV_2410c	Undetectable	129 ± 38	5.1 ±0.2 × 10^3^
MAV_4563	Undetectable	Undetectable	Undetectable
MAV_4563c	Undetectable	102 ± 61	3.1 ±0.4 × 10^3^

*U937 macrophages were infected with the WT 104 or with mutants. The four mutants identified initially were further evaluated for the ability to escape THP-1 macrophages and human monocyte derived macrophages. Viable bacteria in the supernatant were quantified by plating on 7H10 agar plates. We also examined this characteristic using the complement genes (functional), MAV_2235c, MAV_2120c, MAV_2410c, and MAV_4563c. The results obtained were similar to the results using U937 phagocytes*.

MAV_4563 is described as an Mn^++^/Fe^++^ transporter that is probably involved in metal transport in and out of the bacteria. Previous study has demonstrated the importance of intra-phagosome concentration of single elements for bacterial gene expression (Wagner et al., 2005).

MAV_2410 encodes for tatC protein, a component of the tat secretion system. MAV_2235 is an adenylate cyclase and MAV_2120, encodes for a protein of unknown function (Table 1). The phenotype of the mutants was confirmed also by video microscopy comparing to the wild-type bacteria.

Attempts of complementation of all four mutants resulted in the following results: MAV_2235 had its function complemented phenotypically, as well as MAV_2410 and MAV_4563, while MAV_2120 was only partially complemented by the presence of an intact gene in a plasmid. All the mutants grew like the wild-type bacterium in 7H10 agar and behaved similarly to the wild-type bacteria in RPMI-1640 medium.

### MAH survival in macrophages

To determine if the wild-type MAC 104 and the mutants were capable of surviving in macrophages, THP-1 monolayers were infected with the wild-type or the mutant clones and the viable bacteria were quantified after 4 days of infection. As shown in Tables 2A,B, MAH 104 and the clones with mutations in MAV_2235, MAV_2120, and MAV_4563 replicated in macrophages while the clone with MAV_2410 was partially attenuated when compared to the WT MAH 104. The fact that the inactivation of the MAH proteins did not lead to any significant loss of virulence in the macrophage 7 day assay, reinforced the idea that the inactivated proteins are not associated with the classic macrophage virulence phenotype. The level of apoptosis was monitored in all macrophage infection assays and they were approximately the same (data not shown).

**Table 2:** **(A,B) Ability of the identified mutants to survive in THP-1 and human monocyte-derived macrophages in culture**.

**Bacterial Strain**	CFU/ml of macrophage lysate^**^			
	**Time zero day**	**Time 4 days**	**Time 7 days**	**Extra-cellular bacteria 7 days**
**A**				
MAC 104 WT	3.2 ±0.4 × 10^5^	4.0 ±0.3 × 10^6^	6.3 ±0.2 × 10^6^	5.1 ±0.4 × 10^4^
MAV_2235 mutant	3.9 ±0.4 × 10^5^	3.6 ±0.5 × 10^6^	3.9 ±0.4 × 10^6^	Undetectable
MAV_2120 mutant	3.5 ±0.5 × 10^5^	3.7 ±0.3 × 10^6^	4.1 ±0.3 × 10^6^	Undetectable
MAV_2410 mutant	3.3 ±0.3 × 10^5^	1.7 ±0.4 × 10^5^(^*^)	8.2 ±0.4 × 10^4^	7.3 ±0.4 × 10^5^
MAV_4563 mutant	4.1 ±0.4 × 10^6^	4.2 ±0.5 × 10^6^	6.0 ±0.4 × 10^5^	undetectable
**B**				
MAC 104 WT	5.1 ±0.3 × 10^5^	6.3 ±0.4 × 10^6^	8.0 ±0.4 × 10^6^	7.3 ±0.4 × 10^4^
MAV_2235 mutant	4.3 ±0.4 × 10^5^	4.2 ±0.3 × 10^6^	4.7 ±0.2 × 10^6^	Undetectable
MAV_2120 mutant	5.8 ±0.6 × 10^5^	5.9 ±0.4 × 10^6^	6.2 ±0.4 × 10^6^	Undetectable
MAV_2410 mutant	3.8 ±0.3 × 10^5^	5.9 ±0.3 × 10^5^(^*^)	8.4 ±0.3 × 10^4^	1.6 ±0.5 × 10^5^
MAV_4563 mutant	5.6 ±0.5 × 10^6^	4.9 ±0.3 × 10^6^	6.6 ±0.4 × 10^6^	undetectable

* *p < 0.05 compared with WT MAC 104 bacterium*.

** *Number of bacteria/ml of the macrophage lysate*.

### MAH survival in mice

**Figure 2:**
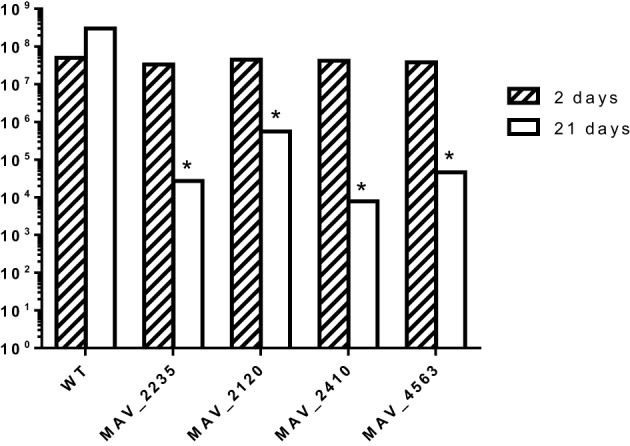
**C57/BL6 black mice were infected with MAH (WT and mutants) and the course of bacterial infection was monitored for 3 weeks**. ^*^p < 0.05 compared with the number of bacteria in mice infected with the WT MAC104.

To determine whether the identified mutants were attenuated in mice, C57BL/six black mice were infected intravenously and 10 mice were harvested after 3 weeks. The spleens were obtained and the viable bacterial number determined after plating. As seen in Figure 2, while the number of bacteria in MAC 104 infected mice increased over time, none of the mutants showed ability to grow in the mouse tissue in a manner comparable to the WT bacteria.

### Potential macrophages partners for identified MAH proteins

To identify potential macrophage partners to the MAH, proteins were cloned into a two-hybrid yeast system and the possible interactions were evaluated. Because the secretion system (tatC) would interact with many mycobacteria proteins, we decided not to carry out the assay using tatC as one of the baits. As described in Table 3, the adenylate cyclase, which contains a secretion peptide, was shown to interact with PNN-serine/arginine-rich proteins (eukaryotic translation terminator, arginine-rich splicing factor 5, SECIS binding protein 2-like) while MAV_4563 interacted with a macrophage ubiquitin specific peptidase 48. In contrast to these putative, secreted MAH proteins, we have no evidence that MAV_2210 can be secreted. MAV_2210 was able to identify and bind a COP9 signalosome subunit (C5N5) that is associated with protein inactivation in the macrophage cytosol.

**Table 3:** **Macrophage targets by identified using the two-hybrid system for the interactions of MAV_2120, MAV_2235, and MAV_4563 encoded proteins**.

**MPH gene name**	**Host gene**
MAV_2210	COP9 signalosome subunit 5 (COPS5)
(Mn^++^/Fe^++^ transporter)	
MAV_2235	PNN- serine/arginine-rich protein
Adenylate Cyclase	
MAV_4563	Ubiquitin specific peptidase 48
Hypothetical protein	

### Host proteins interacting with MAV_2120, MAV_2235, and MAV_4563

**Figure 3:**
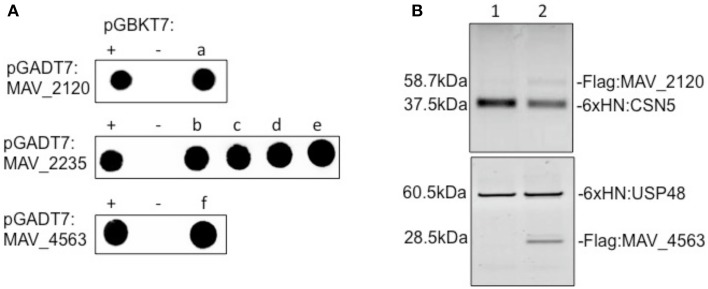
**(A)** The yeast two-hybrid interaction of MAV_2120, MAV_2235, and MAV_4563 proteins with the host target proteins. (a) COP9 signalosome subunit 5 (CSN5), (b) eukaryotic translation termination factor 1 (ETF1), (c) PNN-interacting serine/arginine-rich protein (PNISR), (d) serine/arginine-rich splicing factor 5 (SRSF5), (e) SECIS binding protein 2-like (SECISBP2L), (f) ubiquitin specific peptidase 48 (USP48). **(B)** Co-immunoprecipitation of MAV_2120 and MAV_4563 from *M. smegmatis* cell lysates using recombinant 6xHN:CSN5 and 6xHN:USP48 proteins. Bound proteins were captured on His-columns and visualized by western blotting using 6xHN and Flag antibodies.

To demonstrate direct binding of identified host proteins to *M. avium* proteins, we performed co-immunoprecipitation of Flag-tagged MAV_2120 (Flag:MAV_2120) and MAV_4563 (Flag:MAV_4563) with 6xHN tagged CSN5 (6xHN:CSN5) and USP48 (6xHN:USP48), respectively (Figure 3A). Recombinant 6xHN:CSN5 and 6xHN:USP48 were expressed in the pET system. The Flag:tagged *M. avium* genes were overexpressed in pMV261 and transformed into *M. smegmatis*. Bacteria were lysed at a mid-log growth phase and the cleared protein fraction was incubated with the recombinant host proteins for overnight at 4°C. Recombinant host and bacterial protein mixture were passed through His-columns and bound proteins were visualized with 6xHN and Flag antibodies by western blotting. *M. smegmatis* protein lysate expressing just pMV261 vector was used as a negative control in the co-immunoprecipitation experiment with 6xHN:CSN5 and 6xHN:USP48. As shown in Figure 3B, while no interaction was detected in the control group, purification of recombinant CSN5 and USP48 proteins led to co-purification of MAV_2120 and MAV_4563, respectively, confirming the physical binding between studied host and bacterial proteins.

## Discussion

MAH is a pathogen mostly acquired from environmental sources (Falkinham, 1996; Turenne et al., 2007), although the ability to determine the transmission between humans and the environmental or a human source, is challenging. Once the bacterium crosses the mucosal barrier MAH infects macrophages. It has been shown that the bacteria contain mechanisms that allow for the survival in the phagocytes (Turenne et al., 2007). Many of the survival strategies used by MAH are similar to the ones employed by *M. tuberculosis*, while others are diverse. *M. tuberculosis* infects macrophages, and has been shown in a few models to escape the vacuole as well as the eukaryotic cell (Pieters, 2008; Cambier et al., 2014). This property is made possible by the presence of proteins in the RD1 region of the genome (Pieters, 2008; Cambier et al., 2014). This region, however, is absent in the MAH. MAH after infecting macrophages does not leave the vacuole until macrophage apoptosis is triggered (Early et al., 2011). Following the rupture of the vacuole membrane, the bacterium falls in the cytoplasm of an increasingly diminishing phagocyte cell size (undergoing transformation into apoptotic body), and video microscopy has shown that the encounter between the bacterium with the cytoplasm membrane of the apoptotic macrophage is necessary for existing the macrophage (Early et al., 2011). Therefore, not all the MAH in the cytoplasm are capable to escape the apoptotic macrophage.

To better understand the mechanism associated with the bacterial survival in macrophages and the escape from the phagocytic cell, we partially screened a transposon bank of mutants to identify clones that were incapable of exiting apoptotic cells. The screening resulted in the identification of four genes, one of them tatC, associated with transport of proteins to the outside of the bacterial cell. The other proteins were a putative transport system for Fe^++^ and Mn^++^, that probably is involved in the translocation of intra-vacuolar metals in or out of the bacterium, an adenylate cyclase that contains a signal peptide in its sequence and finally a protein of unknown function.

MAH_2210, a Mn^++^/Fe^++^ transporter, recognized and partnered with the COP9 signalosome subunit 5, which is a cytoplasmic complex associated with the identification of proteins for degradation (Table 3) (Bech-Otschir et al., 2002). MAV_2235 is an adenylate cyclase, that contains a signal peptide. MAV_2235 recognizes PNN-serine/arginine rich proteins, most of them located in nuclear pores or as RNA splicing factors. MAV_4563 is a hypothetic protein that partners with a ubiquitin specific peptidase 48. Apparently, the bacterial proteins identified are mostly involved in interfering or triggering protein degradation as well as affecting post-transcription regulation. COP9 is a protein/complex involved in the activation of the ubiquitin. We have found that a *M. tuberculosis* protein, Rv3354, targets the metalloprtease comain (JAMM) of the signalosome COP9, resulting in suppression of apoptosis (Danelishvili et al., 2014). MAV_2210 interacts with COP9 probably in a different location of the complex, hypothetically to suppress its function. Adenylate cyclases are known to be secreted by pathogens and some of recognized functions resulting from accumulation of cAMP, are associated with inhibition of the phagocyte activity (Rickman et al., 2005; Agarwal et al., 2009). MAV_2235, because it was shown to recognize a protein involved in RNA splicing, may alter the ability of the macrophage to synthesize protein “*de novo*.”

Interestingly, among proteins secreted by the tat secretion system, a few are possible phospholipases (data not shown), which would be expected of proteins that when inhibited would result in the inability to exit macrophages. Our overall findings, however, may point to an unexpected direction. The fact that some of the identified functions of the bacterial proteins seem to be associated with inhibition of protein degradation in the macrophages, we can speculate that the proteins are released in the cytoplasm with the likely goal of inactivating macrophage function, suggesting that the process of exiting from the phagocytic cell is dependent on many other bacterial effectors been exported into the macrophage cytoplasm or potentially nuclear targets. Future studies will certainly address this possibility.

All identified MAH proteins were associated with attenuation of infection in mice, indicating that the process of exiting a phagocytic cell and infecting another cell is quite important as a strategic mechanism of disease.

It is important to mention that the direct link between the escape phenotype and attenuation *in vitro* although suggestive, has not been definitely confirmed yet.

In summary, we described the identification of four MAH proteins that participate in the MAH escapes from macrophages following apoptosis. The inactivation of those proteins have implications for survival within macrophages as well as virulence in mice. Future studies will attempt to characterize their specific role in the process of macrophage escape.

### Conflict of interest statement

The authors declare that the research was conducted in the absence of any commercial or financial relationships that could be construed as a potential conflict of interest.
